# Transient electroretinographic abnormalities that mimic those of *KCNV2* retinopathy: a case report

**DOI:** 10.1007/s10633-021-09828-w

**Published:** 2021-03-18

**Authors:** Chihiro Kaizuka, Takaaki Hayashi, Kei Mizobuchi, Masaomi Kubota, Shinji Ueno, Tadashi Nakano

**Affiliations:** 1 Department of Ophthalmology, Katsushika Medical Center, The Jikei University School of Medicine, 6-41-2 Aoto, Katsushika-ku, Tokyo, 125-8506 Japan; 2 Department of Ophthalmology, The Jikei University School of Medicine, Tokyo, 105-8461 Japan; 3 Department of Ophthalmology, Nagoya University Graduate School of Medicine, Aichi, 466-8560 Japan

**Keywords:** Hyperkalemia, Renal dysfunction, *KCNV2* retinopathy, Cone dysfunction, Full-field electroretinography

## Abstract

**Purpose:**

The purpose of this report was to describe the case of a 68-year-old male patient with stage IV colon cancer who exhibited electroretinographic abnormalities that are similar to those of *KCNV2* retinopathy.

**Methods:**

The patient presenting with photophobia, reduced visual acuity, and poor general conditions, the onset of which occurred ten days before presentation, was examined using fundoscopy, full-field electroretinography, blood tests, and abdominal computed tomography.

**Results:**

The patient’s decimal best-corrected visual acuity (BCVA) was 0.4 in each eye. Fundoscopy showed bull's eye-like maculopathy in both eyes. Electroretinographic findings were similar to the characteristic findings of *KCNV2* retinopathy: Rod electroretinogram showed delayed and preserved b-wave amplitudes; bright-flash electroretinogram showed double troughs of a-waves; b/a ratios shown by bright-flash electroretinogram were higher than those shown by standard-flash electroretinogram; and both cone and 30-Hz flicker electroretinograms showed extinguished responses. His serum potassium level increased to 6.2 mmol/L (normal range 3.6–4.8 mmol/L) owing to hydronephrosis resulting from disseminated carcinoma. After performing an emergency surgery to treat this condition, the serum potassium level immediately decreased to a normal range. Eleven days after presentation, rod and standard/bright-flash electroretinography showed improvement in the implicit time of the rod b-waves and the a-waves. Unexpectedly, the responses recorded by cone and 30-Hz flicker electroretinography became normal. The symptoms and maculopathy disappeared, and his BCVA improved to 1.2.

**Conclusions:**

The abnormal electroretinographic findings might be associated with the transient increase in serum potassium level.

## Introduction

There are two types of photoreceptors—rods and cones—in the human retina. Inherited retinal disorders (IRDs) can selectively affect either rods or cones, leading to the development of progressive rod/rod-cone or cone/cone-rod dystrophies, which are caused by pathogenic gene variants [1–3]. The condition of generalized cone dysfunction is also seen in non-progressive IRDs such as congenital achromatopsia and blue cone monochromacy [1, 2]. Among the IRDs, autosomal recessive *KCNV2* retinopathy, also known as cone dystrophy with supernormal rod responses associated with biallelic variants in the *KCNV2* gene, is a unique form of cone/cone-rod dystrophy [4] that shows a pathognomonic electroretinographic configuration: delayed and normal to subnormal b-wave amplitudes in the dark-adapted (DA) dim flash electroretinogram, flattened/square-shaped trough of a-waves in stronger-flash electroretinogram, relatively higher amplitudes of b-waves (an increased b/a ratio) in strong-flash electroretinogram than in standard-flash electroretinogram, and reduced responses in both light-adapted (LA) photopic and 30-Hz flicker electroretinograms [5–8].

We encountered an elderly patient who exhibited transient and unusual electroretinographic waveforms bilaterally that are similar to those in *KCNV2* retinopathy. The purpose of this report was to describe systemic disease conditions, blood test results, and ophthalmological findings including those of electroretinography of the patient.

## Case presentation

This case report was approved by the Institutional Review Board/Ethics Committee of The Jikei University School of Medicine (approval number: 32-289 10371). This report adhered to the tenets of the Declaration of Helsinki; informed consent was obtained from the patient.

**Fig. 1:**
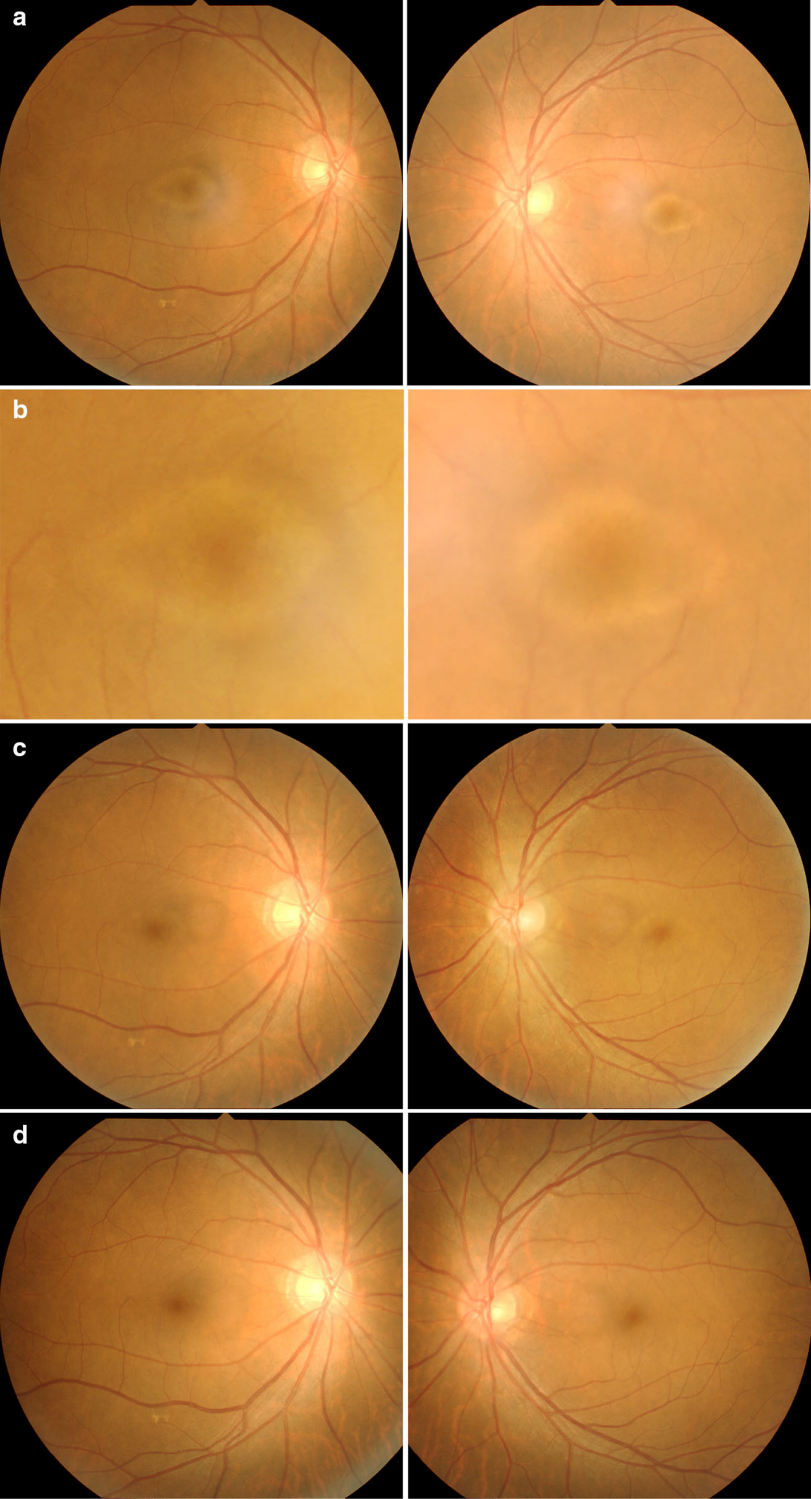
Fundus photographs Photographs in the right eye (left panel) and the left eye (right panel). **a** At presentation: bull's eye-like maculopathy can be observed in both eyes. **b** Magnified photographs of the macular areas at presentation. **c** Eight days after presentation: inconspicuousness of bull's eye-like maculopathy can be observed. **d** One month after presentation: absence of the bull's eye-like maculopathy can be observed

**Fig. 2:**
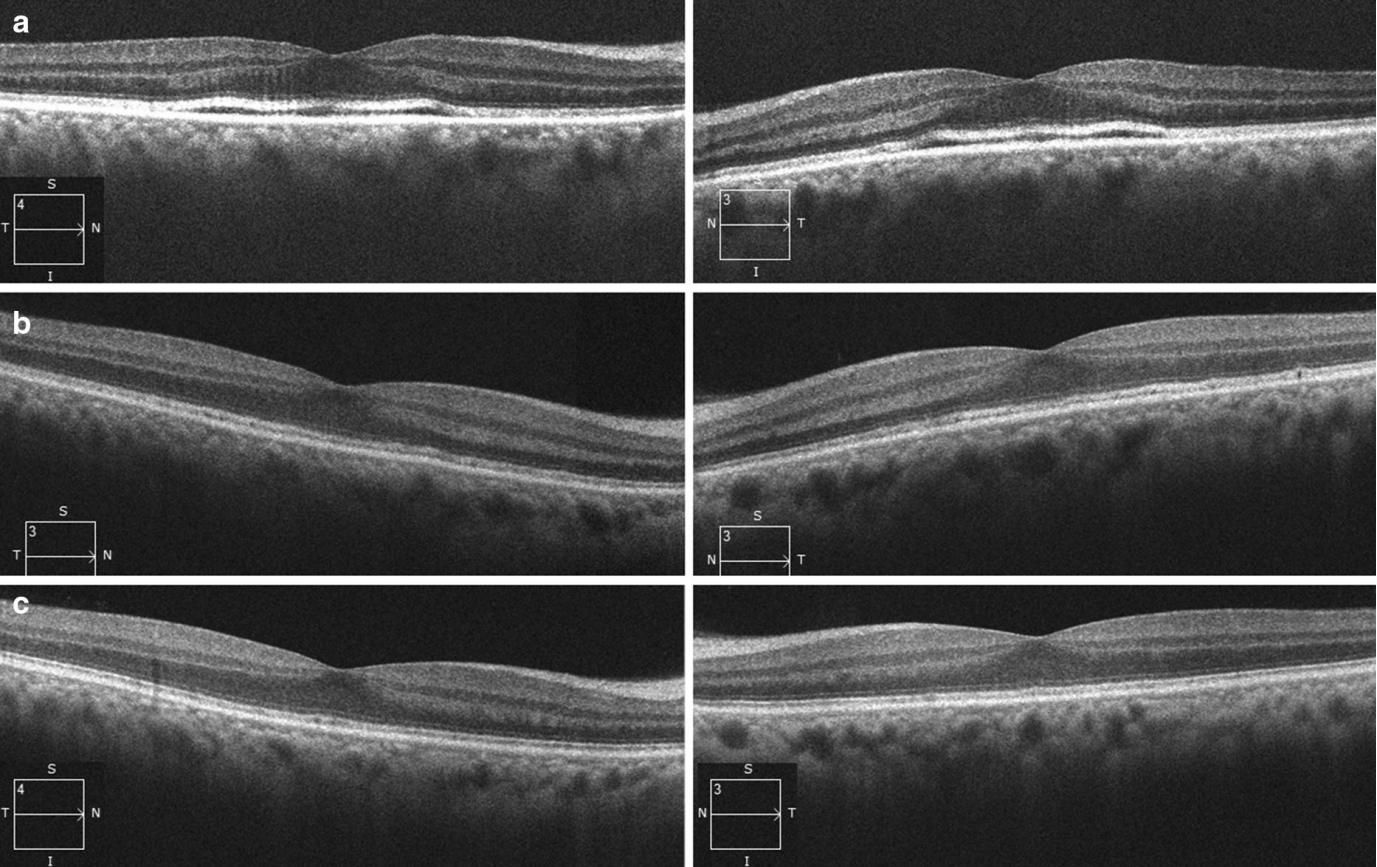
Optical coherence tomography. Horizontal cross-sectional retinal 6-mm images of the right eye (left panel) and the left eye (right panel). **a** At presentation: thickened and elevated ellipsoid zone can be observed at the center of both maculae. **b** Eleven days after presentation: improvement in the ellipsoid zone findings can be observed. **c** One month after presentation: preservation of the improved ellipsoid zone can be observed

**Fig. 3:**
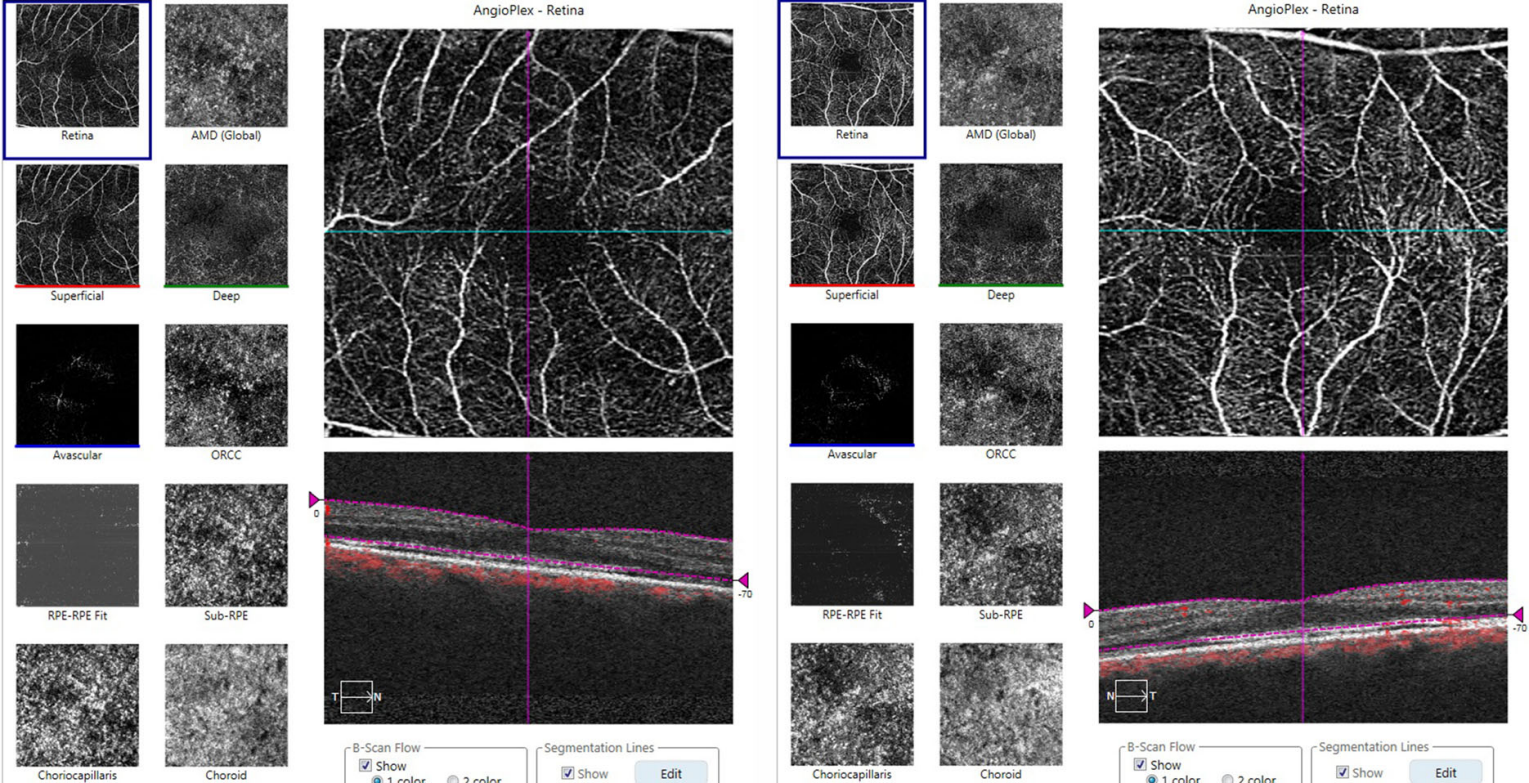
Optical coherence tomography angiography. The 3 × 3-mm *en* face and cross-sectional images of the right eye (left panel) and the left eye (right panel). No flow is detected between the elevated ellipsoid zone and the retinal pigment epithelium in both eyes

**Fig. 4:**
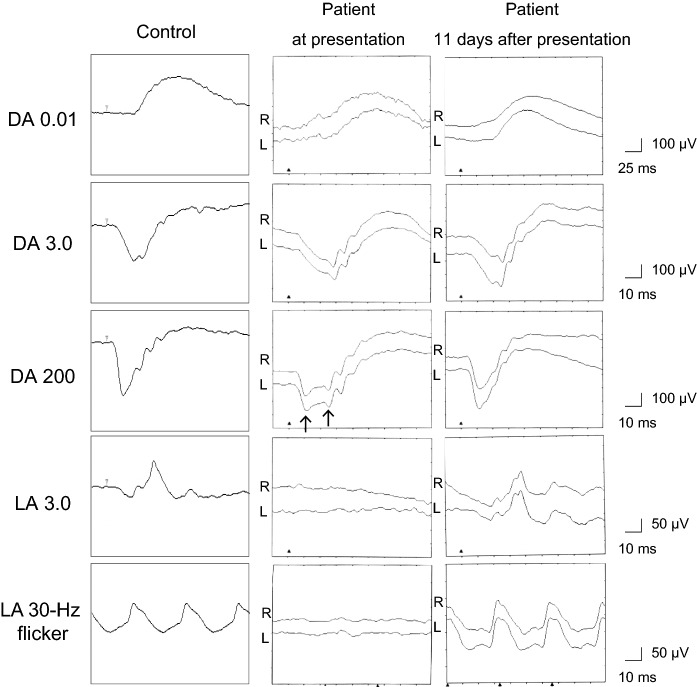
Full-field electroretinograms. Full-field electroretinograms (ERG) in the patient and an age-matched control are shown. At presentation: dark-adapted (DA) 0.01 ERG showing delayed and preserved b-wave responses, DA 3.0 ERG showing delayed and preserved a- and b-waves [b/a ratio: 1.63 in the right eye (RE) and 1.64 in the left eye (LE)], and DA 200 ERG showing decreased a-wave amplitudes with double troughs (arrows) and increased b/a ratios (2.65 in RE and 2.21 in LE, the first trough of the a-waves is adopted). Under light-adapted (LA) conditions: LA 3.0 and 30-Hz flicker ERG show non-recordable responses. Eleven days after presentation: the implicit time of the preserved b-waves in DA 0.01 ERG is normal, while the implicit time of the preserved a-waves in DA 3.0 ERG is still prolonged but shorter than that at presentation. The a- and b-wave amplitudes of DA 200 are normal. The b/a ratios in DA 3.0 are 2.27 in RE and 2.06 in LE, whereas the b/a ratios in DA 200 are 1.76 in RE and 1.49 in LE. The LA 3.0 and 30-Hz flicker ERG become normal

A 68-year-old male patient was referred for assessment of photophobia and reduced visual acuity in both eyes, onset of which was sudden and occurred ten days before presentation to The Jikei University Katsushika Medical Center. He has never had any history of vision problems. One year before presentation, he had undergone sigmoid colectomy for cancer at the Department of Surgery of the same hospital. Liver metastasis was found 7 months after the surgery, and subsequently, the patient was diagnosed with stage IV colon cancer. Later, no medication was prescribed until the presentation. At presentation, we performed comprehensive ophthalmic examinations including electroretinography, although his general condition was poor. His decimal best-corrected visual acuity (BCVA) was 0.4 (Snellen equivalent 20/50) (spherical − 0.25 diopters [D]) in each eye. Slit-lamp examination showed no abnormal findings in the anterior segment and media except for mild age-related cataract. Dilated fundoscopy showed bull's eye-like maculopathy in both eyes (Fig. 1a, b). Horizontal cross-sectional retinal images (6.0 mm) acquired using spectral domain optical coherence tomography (OCT, Cirrus HD-OCT 5000, Carl Zeiss Meditec AG, Dublin, CA, USA) revealed thickened and elevated ellipsoid zone in the center of both maculae (Fig. 2a). OCT angiography 3 × 3-mm scan images (Cirrus HD-OCT 5000) showed that no flow was detected between the elevated ellipsoid zone and the retinal pigment epithelium (Fig. 3), denying presence of abnormal retinal circulation or choroidal neovascularization. At this time, we suspected a retinal disease, such as macular/cone dystrophy or paraneoplastic retinopathy. Full-field electroretinography using a light-emitting diode built-in electrode (LE-4000, TOMEY Corp., Nagoya, Japan) was performed in accordance with the protocols of the International Society for Clinical Electrophysiology of Vision [9], except for a light intensity of 200 cd s/m^2^ (DA 200) instead of DA 10.0 for bright (or strong)-flash electroretinography. The procedure and conditions have been previously reported [10–13]. This ERG system was approved by the Ministry of Health, Labor and Welfare of Japan on September 30, 2002 (approval number: 222AGBZX00211000). All electroretinography responses of the patient were compared with those of previously reported controls (*n* = 23) [12]. The following are electroretinography findings: delayed and preserved b-wave responses in rod (DA 0.01) electroretinogram, delayed and preserved a- and b-waves [b/a ratio: 1.63 in the right eye (RE) and 1.64 in the left eye (LE)] in standard-flash (DA 3.0) electroretinogram, and decreased a-wave amplitudes with double troughs and increased b/a ratios (2.65 in RE and 2.21 in LE, the first trough of the a-waves was adopted) in bright-flash (DA 200) electroretinogram. Under LA conditions, cone (LA 3.0; background, 30 cd/m^2^) and 30-Hz flicker (background, 30 cd/m^2^) electroretinography was performed, which showed non-recordable responses (Fig. 4), demonstrating some kind of cone dysfunction syndrome.

**Fig. 5:**
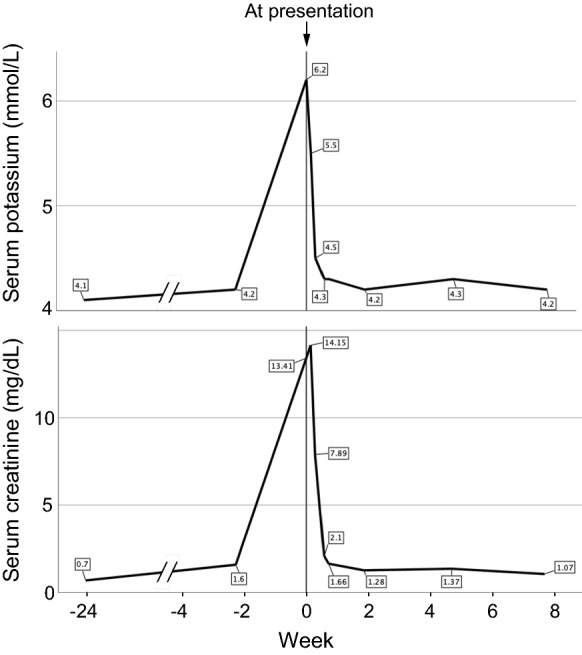
Time course of serum potassium and creatinine levels. At presentation, serum potassium and creatinine levels increased to 6.2 mmol/L (normal range 3.6–4.8 mmol/L) and 13.41 mg/dL (normal range 0.65–1.07 mg/dL), respectively. After emergency surgery on the day after presentation, the potassium and creatinine levels were reduced to normal and nearly normal levels within a couple of days, respectively

On the same day, blood tests and abdominal computed tomography were performed in the Department of Surgery. On the basis of the findings of these examinations, he was diagnosed with hydronephrosis resulting from ureteral obstruction secondary to disseminated carcinoma. His venous serum potassium and creatinine levels increased to 6.2 mmol/L (normal range 3.6–4.8 mmol/L) and 13.41 mg/dL (normal range 0.65–1.07 mg/dL), indicating renal dysfunction. Preoperative arterial blood gas showed pH 7.33 (normal range 7.35–7.45), decreased partial pressure of arterial carbon dioxide 27.3 mmHg (normal range 35–45 mmHg), increased partial pressure of arterial oxygen 117 mmHg (normal range 85–105 mmHg), and decreased bicarbonate concentration 13.8 mmol/L (normal range 23–28 mmol/L). He underwent emergency surgery at the Department of Urology on the day after presentation. Time courses of venous serum potassium and creatinine levels are shown in Fig. 5. The graph was made using IBM SPSS Statistics version 26.0 (IBM Corp, Armonk, NY, USA). After the surgery, the potassium and creatinine levels were reduced to normal and nearly normal levels within a couple of days, respectively.

We followed up the patient for three months. Eight days after presentation, his BCVA improved to 1.0 (Snellen equivalent 20/20) bilaterally. The configuration of bull's eye-like maculopathy was inconspicuous (Fig. 1c). Eleven days after presentation, electroretinography was performed. At this time point, venous serum potassium levels were within the normal range (Fig. 5). The implicit time of the preserved b-waves in DA 0.01 electroretinogram was normal, while the implicit time of the preserved a-waves in DA 3.0 electroretinogram was still prolonged but shorter than that at presentation (Fig. 4). The a- and b-wave amplitudes in DA 200 electroretinogram were normal. The b/a ratios in DA 3.0 electroretinogram were 2.27 in RE and 2.06 in LE, whereas the b/a ratios in DA 200 electroretinogram were 1.76 in RE and 1.49 in LE. The responses of LA 3.0 and 30-Hz flicker electroretinography became normal. Morphologically, the ellipsoid zone findings were also improved (Fig. 2b). One month later, fundoscopy showed disappearance of the bull's eye-like maculopathy (Fig. 1d). At this timepoint, his BCVA was 1.2 (Snellen equivalent 20/16.7) in each eye, and photophobia had disappeared. Furthermore, the improved ellipsoid zone findings were preserved (Fig. 2c). At the last visit 3 months after presentation, his BCVA was maintained at 1.2 in each eye.

## Discussion

In this report, we described clinical findings of a patient who exhibited transient and unusual electroretinographic findings including extinguished photopic responses.

The abnormal electroretinographic findings (Fig. 4) were similar to those of *KCNV2* retinopathy [5–8]. Unexpectedly, the responses of LA 3.0 and 30-Hz flicker electroretinography became normal only 11 days after presentation (Fig. 4). As for diagnosis, at least, macular diseases such as macular/cone dystrophy and drug-induced and paraneoplastic retinopathies were ruled out.

Although we could not determine the exact reason why the patient showed transient electroretinographic abnormalities, we focused on the presence of hyperkalemia because except for high levels of venous serum creatinine and potassium concentration at presentation, there were no differences between blood test results at presentation and at about 2 weeks before presentation (Fig. 5). In the literature, we found that there have been two important reports about a relationship between serum potassium concentration and electroretinographic b-wave amplitude [14, 15]. Levodopa (also called L-dopa), a dopaminergic drug, is the most commonly prescribed medication for Parkinson's disease. In 1979, Filipova et al. have reported that b-wave amplitudes of DA flash (2.0 J in intensity) electroretinography in patients with parkinsonism treated with L-dopa are significantly greater than those in controls [14]. Three years later, the same group has demonstrated that not only b-wave amplitudes, but also serum potassium levels are abnormally high at 60 min after L-dopa administration [15]. At 180 min after L-dopa administration, decrease in b-wave amplitude and normalization of potassium concentration have been observed [15]. Conversely, a previous report showed that metoclopramide, one of the dopamine antagonists, decreases serum potassium concentration in healthy volunteers [16]. These data suggest that there may be a positive relationship between b-wave amplitude and serum potassium level. Unfortunately, the abovementioned two reports have not mentioned implicit time/amplitude/waveform configuration of a-waves or b/a ratio [14, 15]. In our patient, the b/a ratios in strong-flash (DA 200) electroretinogram at presentation were greater than those recorded 11 days after presentation and in the control(s) (Fig. 4). The serum potassium level was abnormally high (6.2 mmol/L) at presentation, which decreased immediately to 4.5 mmol/L at 2 days after presentation (one day after the surgery) (Fig. 5). Thereafter, the potassium levels were maintained within the normal range during the follow-up. Therefore, the electroretinographic abnormalities of the patient are likely to be associated with the transient increase in serum potassium levels but not persistent hyperkalemia. However, since decreased bicarbonate concentration was detected in the preoperative arterial blood gas test, the possibility that the decreased bicarbonate might impact on electroretinographic responses cannot be denied.

The *KCNV2* gene encodes the voltage-gated potassium channel subunit Kv8.2 [4], which is expressed in both rods and cones [17]. *KCNV2* retinopathy is considered to be the first human disorder associated with potassium channel dysfunction to affect the visual pathway of the retina [4]. The reason why *KCNV2* retinopathy predominantly affects cones remains to be unresolved. It was hypothesized that the distribution of Müller cells in the retina could be an important factor because their regulatory and buffering effects on the extracellular potassium ions are missing especially in the cone-rich or macular area, making cones more vulnerable [18]. With regard to electroretinographic components, not only bipolar cells but also Müller cells are involved in the generation of the b-wave of strong-flash electroretinography [19–22]. In our patient, the imbalance between intracellular and extracellular potassium levels around the photoreceptors might have resulted from the transient hyperkalemic condition. Collectively, it was suspected that the rapid elevation of serum potassium concentration might be associated with generalized cone dysfunction, leading to the unusual electroretinographic findings that were similar to those of *KCNV2* retinopathy. However, it should be noted that the mechanism how the hyperkalemic condition predominantly influences cones remains to be solved.

In conclusion, we described clinical findings of an elderly patient who exhibited transient electroretinographic abnormalities that are similar to those of *KCNV2* retinopathy. The detailed clinical course and blood test results suggest that the electroretinographic findings might be associated with the transient increase in serum potassium level.

## Declarations

### Statement on the welfare of animals

This article does not contain any studies with animals performed by any of the authors.
